# Language-specific approaches to reduce perioperative stress and anxiety related to anaesthesia for patients with limited English proficiency: A narrative review

**DOI:** 10.1177/17504589251316744

**Published:** 2025-02-13

**Authors:** Kanwarpreet Kaur Dhaliwal, Sherry Sandhu, Nitasha Puri, Amolpreet Toor

**Affiliations:** 1 Faculty of Medicine, The University of British Columbia, Vancouver, British Columbia, Canada; 2 Fraser Health Authority, Surrey, British Columbia, Canada; 3 Department of Anesthesiology, Pharmacology, and Therapeutics, The University of British Columbia, Vancouver, British Columbia, Canada

**Keywords:** Perioperative anxiety, Anaesthesia, Informed consent, Language-specific approaches

## Abstract

Many patients experience perioperative anxiety due to a variety of different reasons. Essential processes of shared decision-making and informed consent may help to mitigate anxiety, yet language barriers may hinder this particularly in diverse patient populations. As such, language-specific approaches in anaesthesia care play a crucial role in reducing perioperative stress and anxiety among patients with limited English proficiency. This review examines which methods during anaesthetic assessments and shared decision-making processes enable anaesthetists to communicate effectively with patients who have limited English proficiency and thereby reduce perioperative stress. Findings suggest that collaborating with patients in their native language significantly reduces anxiety and improves understanding, while transcreation – culturally adapted translation – enhances the effectiveness of communication. To decrease perioperative anxiety among populations with limited English proficiency and improve surgical outcomes, it is important to enhance anaesthesia-focused training for interpreters, increase diversity in the anaesthesia field, and develop culturally relevant patient education materials.

## Introduction

Previous studies have indicated that many patients experience fear of the risks related to general anaesthesia, resulting in perioperative stress and anxiety (Friedrich et al 2022, Mavrogiorgou et al 2023). Perioperative anxiety has been associated with increased experiences of postoperative pain (Ali et al 2014, Inal et al 2021) and longer recovery times (Schaal et al 2020). Studies conducted across cardiology, obstetric, and major general surgery settings have suggested that perioperative anxiety may also lead to increased morbidity and mortality (Aspalter et al 2021, Takagi et al 2017). Furthermore, previous studies have found that perioperative anxiety can necessitate increased induction dosing regimens (Ahmetovic-Djug et al 2017, Inal et al 2021, Kil et al 2012, Tadesse et al 2022) and intraoperative haemodynamic instability (Inal et al 2021).

Non-pharmacological mechanisms to reduce perioperative stress and anxiety include cognitive behavioural therapy, music therapy, and patient education (Wang et al 2022). The essential process of shared decision-making, in which patients and their families are actively involved in the process of making decisions with health care providers (Faiman & Tariman 2019), could also be an opportunity to mitigate perioperative anxiety. Shared decision-making often includes elements of informed consent, which is a process that consists of physicians explaining risks, benefits, and details of a procedure or intervention to a patient prior to them agreeing to receive it (Cocanour 2017, King & Moulton 2006, Richardson 2013). Older studies indicate that improved informed consent can play a role in improving patient satisfaction and knowledge, while also reducing perioperative stress and anxiety (Kinnersley et al 2013, West et al 2014). Furthermore, as practice has evolved beyond informed consent towards the collaborative process of shared decision-making, it has been noted that patients and providers alike feel more satisfied (de Mik et al 2018, Shinkunas et al 2020). However, some scholars have noted serious limitations to obtaining informed consent and shared decision-making in surgical specialities, including lack of time, and delegating informed consent to junior members of the medical team (Wang et al 2025). Furthermore, lack of informed consent is a common factor for malpractice litigation among surgical specialties (Custer et al 2017, Grauberger et al 2017, Shlobin et al 2020). Similarly, scholars have noted that in some jurisdictions, anaesthetists have lagged in embracing patient-centred shared decision-making (Sturgess et al 2019). Anaesthetists have a responsibility to communicate effectively with patients during informed consent and shared decision-making processes, and therefore, the inability of an anaesthetist to do so may result in increased anxiety and dissatisfaction among patients as well as impede accurate and valid preoperative anaesthesia assessment (Cheng et al 2004, Nagrampa et al 2015, Rai et al 2019, Shapeton et al 2017).

Effective communication during the informed consent and shared decision-making processes may be more difficult for anaesthetists who serve diverse patient populations in which there are varying literacy rates and abilities to speak, read, and understand English (Quadri & Saunders 2003). It has been shown that language concordance and use of professional medical interpreters can improve care and informed consent processes among patients with limited English proficiency (LEP) (Lee et al 2017, Luan-Erfe et al 2023, Molina & Kasper 2019). The use of language translators in anaesthesia to communicate with patients has been heavily documented (Luan-Erfe et al 2023); however, issues that arise when utilising language translators include under-resourced interpreting services, reliance on relatives as language translators, and differences between what the provider wishes to inform versus what the translator relays to the patient (Quadri & Saunders 2003, Shapeton et al 2017). It is apparent that the issues with the interpreter–provider interaction may impact the quality of language concordance for patients with LEP (Shapeton et al 2017).

Some academics have looked at structural workplace factors that might improve patient–provider communication, such as ethnocultural and language diversity in the anaesthesia workforce (Estime et al 2021). However, recent studies have shown that anaesthesia residency programmes in the United States have structural factors in place that are preferentially favouring Caucasian applicants over Black and Hispanic (Bradley et al 2022, Rosales et al 2024).

Overall, it is apparent that anaesthetists must have the ability to effectively communicate with patients who have LEP in order to obtain authentic informed consent, ensure an accurate preoperative assessment, and thereby reduce perioperative stress and anxiety. Currently, the evidence surrounding factors and processes that can alleviate perioperative stress is varied and scoping in nature. As diversity among patient populations increases, it is clear that a more nuanced understanding regarding anaesthetists’ role, language services, and representation when delivering patient education is pivotal to achieving patient satisfaction and reducing fear of surgery and anaesthesia. Thus, this narrative review explores the following question: *Which methods, processes, or approaches to care and shared decision-making enable anaesthetists to communicate effectively with patients who have LEP, and thereby reduce perioperative stress and anxiety levels?*

## Methods

Narrative reviews are a useful form of knowledge synthesis to provide a general summary with interpretation and critique of existing literature (Sukhera 2022). Whereas systematic reviews are focusing on a narrow question in a specific context (Sukhera 2022), narrative reviews are broader in nature to provide an overall summary with interpretation and critique (Sukhera 2022). In this article, a literature search was conducted using Ovid MEDLINE, evaluating racial experiences with anaesthesia, perioperative and postoperative complications, and patient education. Search keywords included Anaesthes* (‘anaesthesia’ or ‘anaesthesia general’ or ‘anaesthesiology’); Raci* (‘racial groups’ or ‘ethnicity’); Patient Education; Educat* (‘educational status’ or ‘education’ or ‘medical’ or ‘curriculum’ or ‘education’ or ‘undergraduate’); Language Translation (‘language’ or ‘survey and questionnaire’ or ‘translation exp’). The inclusion criteria for the initial search were as follows:

- Any type of study methodology, not limited to meta-analyses, scoping reviews, reports and grey literature, randomised control trials, cohort and/or case–control studies, and case reports.
- Patients of any age.
- Studies focused on visible minorities and racialised populations, not limited to South Asian, Hispanic, Indigenous, as well as populations who are non-English-speaking in English-speaking countries.
- English language.

Exclusion criteria were as follows:

- Studies that had administered anaesthesia, but did not explore anaesthesia as the focus.
- Pharmacological trials regarding anaesthesia.

This search generated 22 abstracts. Fourteen of the studies fit the inclusion criteria as decided by one researcher. A citation search was also conducted on the relevant manuscripts and this generated four additional manuscripts. Overall, 18 manuscripts were interpreted and critically assessed by EQUATOR guidelines when able. The most relevant studies were summarised into the narrative results, but to reduce redundancy some studies were excluded.

## Results

The studies examined for this review revealed three main concepts around approaches to care for LEP populations undergoing anaesthesia. These are as follows:

1. Education in a patient’s native language and rooted in the patient’s cultural context can minimise stress, improve patient satisfaction, and improve health outcomes.
2. It is important to ensure that patients understand the scope and role of anaesthetists, and if possible, see diversity reflected in the anaesthetic workforce.
3. Language interpreters, when used in clinical settings to provide translation and transcreation services, are highly effective; however, the current utilisation of language translators in anaesthetic settings is minimal or incorrect.

### Patient education in native language

Lower perioperative knowledge is associated with increased perioperative anxiety (Kureshi et al 1995). Cheng et al (2004) conducted a prospective study to elucidate barriers to effective perioperative communication between Indigenous Australians and anaesthetists. In this study, 82.3% of the Indigenous Australian population did not speak English as their first language.

The language barrier culminated into a lack of understanding between the patients and their providers, where 28.7% of patients felt that they did not understand their anaesthetist’s explanation.

Conversely, a study was conducted by de Armendi et al (2014) in which Spanish-speaking parents were provided with a Spanish video about the risks and benefits of anaesthesia and then their anxiety was measured via the Amsterdam Preoperative Anxiety and Information Scale (APAIS). Results concluded that Hispanic parents who viewed the pre-anaesthesia Spanish video had a 3-point decrease on the APAIS scale compared to patients who did not watch the video (de Armendi et al 2014). Although the statistical significance could not be verified due to a low sample size, the trend was evident.

Outside of the field of anaesthesia, there is evidence to suggest that patient education in one’s native language, especially for those who have LEP, improves outcomes. Reaume et al (2024) conducted a population-based cohort study of home care residents living in Ontario, Canada and found that for patients who had a language-concordant family physician, there were fewer emergency department visits (53.1% vs. 57.5%; p < 0.01), fewer hospitalisations (35% vs. 37.6%; p < 0.01), and less mortality (14.4% vs. 16.6%; p < 0.01).

### Anaesthesiology lacks diversity

LEPs are over-represented in racialised populations, and research has found that race concordance among patients and providers improves communication, perception of care, and better health outcomes (Moore et al 2022). Data have shown that there is a lack of understanding regarding the role of anaesthetists among racialised populations, and that the lack of diversity among anaesthetists may have culminated into a lack of trust. Findings from a cross-sectional survey showed that 50% of patients did not know the years of medical school or training required of anaesthetists, and 24.7% of patients elected ‘don’t know’ to a question asking ‘who is responsible for monitoring your vital signs throughout surgery?’ (Nagrampa et al 2015). This lack of knowledge pertaining to the role of anaesthetists was hoped to be compensated by 83% of patients who reported that meeting the anaesthetist before surgery was necessary (Nagrampa et al 2015). Furthermore, 87% of participants wanted the anaesthetists to discuss their anaesthesia in detail (Nagrampa et al 2015). The data collected indicated that trust in an anaesthetist was significantly related to a respondent’s knowledge of anaesthesia (Nagrampa et al 2015). The lack of trust by the Hispanic population was further corroborated by Stepanikova et al (2006), who discovered that minorities in general have lower scores on multivariate models on variables that measure trust.

Research has deduced that greater diversity in the health care team will improve the accuracy of clinical decision-making leading to higher patient satisfaction, trust, and improved patient outcomes (Gomez & Bernet 2019). More specifically, for LEP and racialised populations, having an anaesthetist who may be able to speak their language or come from a similar background may further increase trust in the provider. In the United Kingdom, the Royal College of Anaesthetists does not publish data on the ethnic composition of anaesthetists (Royal College of Anaesthetists 2020). However, the General Medical Council reported that in 2021, 76.8% of anaesthetists and intensivists were Caucasian graduates, compared to 20.9% from ethnic minority backgrounds (General Medical Council 2023). This disparity has been consistent since 2007, with Caucasian graduates consistently entering these fields at a significantly higher rate than their ethnic minority counterparts (General Medical Council 2023). Data collected by Nafiu et al (2020) highlighted that while the number of paediatric anaesthetist fellows in the United States increased at an average rate of nine fellows per year, the proportions of black and other minority trainees remained low at rates of 5.0%–6.5% for Black and other minorities at 8.2%–8.5%. This finding was further corroborated by Sesi et al (2023), who found that cardiothoracic anaesthesia fellowship programmes continue to be male-sex dominant and of white racial representation. The CASPer score, which is a situational judgement test utilised by anaesthesia residencies across North America, is a potential structural factor. Rosales et al (2024) found that on a CASPer test for anaesthesia residency programmes, z-scores among applicants of white candidates (0.18) scored significantly higher than Black (0.57) and Hispanic (0.52). Furthermore, Bradley et al (2022) conducted questionnaires to deduce applicant’s preferences for certain anaesthesia residency programmes over others, and data concluded that medical trainees in underrepresented groups desire to train at a programme that is diverse. For example, 38% of males and 25% of females reported that they did not apply to programmes that had a lack of diversity (Bradley et al 2022) Overall, the lack of diversity in the anaesthetic work force in North America and the United Kingdom may also contribute to lack of trust and understanding among LEP patients.

### Interpretation, translation, and transcreation

The role of language translators in the care of non-English-speaking populations undergoing anaesthesia is clear; Cheng et al (2004) found that Anaesthesia Interpreter Services (AIS) improved communication 66.7% of the time when it was used. More broadly, it is clear that translation services improve health care overall, as suggested by the findings of a systematic review and qualitative analysis conducted with interpreters (Karliner et al 2007, Wu & Rawal 2017).

Despite this, interpretation is underutilised, and existing translators lack training in the area of anaesthesia. In a study conducted in Boston, 96% of interpreters who regularly provided services to the anaesthesia department denied receiving anaesthesia-specific training (Shapeton et al 2017). The same study found that there were concerns raised by anaesthesia providers regarding the fidelity of translation (Shapeton et al 2017). Research done by Deol (2023) corroborated these findings; an interview was conducted with an executive from a provincial health authority’s language service department within British Columbia, Canada, in which the executive stated that despite administering a written language test, there is no mandate for official training or certification among medical interpreters.

In addition, interpreter services are not utilised as often as they should be. Cheng et al (2004) concluded that out of a pool of ten patients who needed language translation, the AIS service was not used due to: being difficult to organise in five patients, service unavailability in three patients, and no explanation given for two patients. Cheng et al (2004) highlighted that anaesthetists would not consider providing anaesthesia to anyone who spoke little or no English unless in the case of an extreme emergency, but in this study over a 2-month period, 29 patients underwent anaesthesia without an interpreter. More recent studies have found that interpreter usage remains potentially underutilised for surgical patients with LEP (Cevallos et al 2024).

Alongside interpretation services, there is also evidence to suggest that the process of transcreation and translation with cultural context can improve engagement and outcomes for patients with LEP. Transcreation is an adaptation process that enhances cultural relevance to translation by addressing context, concepts, needs, goals, and language (Nelson et al 2024). Meade et al (2023) articulated that the role of transcreation in health equity is that it appreciates cultural knowledge as a means of requirement for communication. Cheng et al (2004) highlighted this phenomenon in the case of an Australian Indigenous population; Indigenous culture has extensive knowledge of healing and theories of disease that are distinct from the Western biomedical model, and if the patient education is not delivered in with an emphasis on the culture of the patient, then they may not recognise that the quantitative rates like complication rates of anaesthesia have little meaning for Indigenous patients. Furthermore, Cheng et al (2004) found that many English words do not have direct equivalents for the over 300 Indigenous languages/dialects present in the Northern Territory.

Although this has not been further established in the anaesthesia literature, conclusions from other areas of health care may be applicable to the anaesthesia setting. An example of transcreation in care was conducted by Nelson et al (2024), who conducted semi-structured interviews with a Latinx Spanish-speaking population undergoing cancer treatment to produce a guide about cancer care. Nelson et al (2024) used an English template which was directly translated into Spanish and then was further refined for cultural attributes by interviewing patients about values regarding clinicians, personhood, and health care preferences. The guide was then assessed for understandability, acceptability, relevance, and responsiveness within the Latinx community. Another study conducted by Fortney et al (2024) piloted test study material that were transcreated from English to Spanish with the assistance of a bilingual community advisory board with Spanish-speaking parents of neonatal intensive care unit (NICU) infants. Fortney et al (2024) found that after transcreation, the internal reliability of study instruments ranged from good to excellent and participants reported that the study materials were not offensive and did not make them feel offended.

## Discussion

This narrative review explored what is currently known about methods to reduce perioperative stress in populations with LEP undergoing anaesthesia. Given the vast evidence that persists regarding the benefits of providing patient education in one’s native language, the strengths and limitations of AIS, and the gaps in patient knowledge about anaesthetist and their role in patient care, it is clear that a more focused and nuanced approach to addressing perioperative anxiety among LEP populations is required.

Despite the limited information available, it appears that in order to reduce perioperative anxiety for LEP populations, it is worthwhile to consider recruitment of more diverse anaesthetists, improved education about the role of anaesthetists during informed consent, and provision of patient education materials subjected to a transcreation process. In addition, interpreters who are providing care for the anaesthesia department should have distinct training pertaining to anaesthesia rather than general care. Similar to patient education resources, interpreter training should also accept a transcreation approach (Meade et al 2023). By having interpreter services well nuanced in the role of cultural understanding, they will ensure that the knowledge they are disseminating is presented in a way that will identify with the patient.

### Limitations

This narrative review has limitations which should be acknowledged. The scoping review was conducted by one researcher. As a result of this, there is a reliance on a limited number of studies which restricts the generalisability of the findings. The included studies predominantly focused on specific racial and ethnic groups like Indigenous Australians and Hispanics, which may not represent the full diversity within the patient population. The review also primarily relies on English-language studies, potentially overlooking relevant research published in other languages. In addition, the search strategy may have missed some relevant studies due to the limitations of the selected databases and keywords. The review also does not account for potential publication bias, where studies with positive findings are more likely to be published than those with negative or null results.

### Future research directions

There is a clear need for educational resources that increase knowledge about the role of anaesthetists among racialised and LEP folks. In addition, in-depth studies about where patients seek their information regarding anaesthesia or anaesthetists might inform clinical implications. This may inform studies on patient’s views of the effectiveness of communication. There should also be exploration on the role of effective shared decision-making within anaesthesia impacting patient outcomes among LEP populations. Finally, there should be research conducted on the impact of transcreation versus translation in interpreter services and the lasting impact on patient outcomes.

## Conclusion

This narrative review has highlighted the critical importance of patient education in their native language, knowledge surrounding the role of anaesthetists, and the use of language translators in reducing perioperative stress and anxiety among populations with LEP. The findings suggest that effective communication and education tailored to a patient’s cultural background can significantly enhance patient satisfaction and trust in anaesthesia care.
